# Pediatric Gastroenterologists' Practice Styles and Compliance With European/North American Helicobacter pylori Guidelines

**DOI:** 10.7759/cureus.99707

**Published:** 2025-12-20

**Authors:** Ashwin Agrawal, Anupama Chawla, Héctor E Alcalá, Lesley Small-Harary

**Affiliations:** 1 Pediatric Gastroenterology, RWJBarnabas Health, Long Branch, USA; 2 Pediatric Gastroenterology, Rutgers Robert Wood Johnson Medical School, New Brunswick, USA; 3 Pediatric Gastroenterology and Nutrition, Stony Brook Children's Hospital, Stony Brook, USA; 4 Behavioral and Community Health, University of Maryland School of Public Health, College Park, USA

**Keywords:** guideline, helicobacter pylori, h. pylori, pediatric gastroenterology, pediatrics, physician guideline adherence, treatment choices, upper endoscopy

## Abstract

Objective

This study aims to determine pediatric gastroenterologists’ adherence to the management, diagnosis, and treatment of *Helicobacter pylori* (*H. pylori*) per the joint European Society for Pediatric Gastroenterology, Hepatology and Nutrition (ESPGHAN)/North American Society for Pediatric Gastroenterology, Hepatology and Nutrition (NASPGHAN) guidelines for *H. pylori *in children (update 2016).

Methods

A survey was sent to the pediatric gastroenterology listserv to assess the knowledge of guidelines, workup, and treatment of *H. pylori* infection. The survey was open to respondents between 4/13/2022 and 5/12/2022.

Results

A total of 302 individuals completed the survey, representing 25 countries. The most ordered test for *H. pylori* was the stool antigen test (n = 151, 56.62%), followed by upper endoscopy (n = 97, 32.12%). Based on positive non-invasive test, 123 (40.72%) providers would never or not often treat *H. pylori*.

Most providers treat *H. pylori* on histopathology in the absence of peptic ulcer disease. The majority of providers did not send *H. pylori* susceptibility testing. Most waited for the appropriate time after discontinuing antibiotics/anti-acid medication prior to testing for *H. pylori.*

In the absence of susceptibility testing, 121 (40.5%) providers utilized proton pump inhibitors, amoxicillin, and clarithromycin to treat *H. pylori* compared to 79 (26.4%) providers who utilized proton pump inhibitors, high-dose amoxicillin, and metronidazole.

Conclusion

Areas where gastroenterologists adhere to the 2016 guidelines include waiting time after stopping antibiotics/anti-acid therapy and testing for eradication.

Pediatric gastroenterologists had less adherence with the use of non-invasive testing for the diagnosis of *H. pylori*, treatment based on non-invasive test, diagnosis of *H. pylori* by endoscopy, utilizing culture and susceptibility testing, and antibiotic regimen used to treat *H. pylori*.

Our survey results indicate that pediatric gastroenterologists require further education, especially now that the updated 2024 guidelines have been released.

## Introduction

*Helicobacter pylori* (*H. pylori*) is an infection acquired by humans and can cause significant morbidity. It is a well-known cause of peptic ulcer disease and gastritis in both children and adults. If left untreated, it can lead to gastric cancer, primarily in adults [1]. The global prevalence of *H. pylori* in children is estimated to be 33% [2]. Given the high prevalence and complications associated with *H. pylori* infection, there has been an international effort to create consensus-based guidelines for the evaluation and treatment of *H. pylori*. A widely accepted guideline was jointly created by the North American Society for Pediatric Gastroenterology, Hepatology and Nutrition (NASPGHAN) and the European Society for Pediatric Gastroenterology, Hepatology and Nutrition (ESPGHAN) [1].

A North American survey conducted in 2003 assessed the practices of pediatric gastroenterologists in regards to *H. pylori* testing and treatment, which showed that practices varied widely [3]. A study in 2019 of general pediatricians in Israel showed that only about one-third of surveyed pediatricians followed the ESPGHAN/NASPGHAN guidelines to diagnose and treat *H. pylori* infection [4]. More recently, in 2021, a pediatric quaternary care center reported low adherence of its providers to the ESPGHAN/NASPGHAN *H. pylori* guidelines [2]. However, to date, there has been no national or international survey to assess the adherence and practice styles of pediatric gastroenterologists with regard to the management of suspected or confirmed *H. pylori* infection.

This study aims to determine pediatric gastroenterologists’ adherence to the management, diagnosis, and treatment of *H. pylori* as per the “Joint ESPGHAN/NASPGHAN Guidelines for the Management of Helicobacter pylori in Children and Adolescents (Update 2016)” [1]. This study aims to determine which practice areas (diagnosis, endoscopy, and treatment) pediatric gastroenterologists are more likely to follow regarding *H. pylori* in children. Though there has been a recent update to the guidance in 2024, the majority of the new recommendations reaffirmed the 2016 recommendations, with only a significant change of recommendation being that the antimicrobial regimens were changed [5]. Given that the vast majority of the recommendations in the 2016 versus the 2024 guidelines are the same, this study helps provide insight into pediatric gastroenterology practice regarding *H. pylori* infections.

## Materials and methods

Ethics statement

This research study was approved by Stony Brook University’s Institutional Review Board (IRB2022-00156).

Survey design

The survey instrument was designed in Stony Brook University’s REDCap (Vanderbilt University, Nashville, TN), a secure, Health Insurance Portability and Accountability Act (HIPAA)-compliant, web platform for building and maintaining online databases and surveys. The survey instrument was anonymous, and no identifying information was collected.

Survey measures

The survey was designed to assess practices and adherence to the NASPGHAN/ESPGHAN 2016 *H. pylori* guidelines. The survey consisted of demographic questions, questions assessing knowledge of guidelines, and questions assessing pediatric gastroenterologists’ practices regarding workup and treatment of suspected *H. pylori* infection. The survey can be found in the Appendices. The survey was assessed by 11 pediatric gastroenterology providers (nine physicians and two nurse practitioners) to assess language and understandability and ensure the survey would make sense to a broad range of gastroenterologists. The 11 pediatric gastroenterology providers also assessed the survey instrument for readability, ensuring the questions were clear, relevant, and easy to understand. All 11 pediatric gastroenterology providers treat patients with *H. pylori* and are knowledgeable about *H. pylori*. Feedback from 11 pediatric gastroenterology providers was used to improve the survey instrument, which was then reassessed by the 11 providers prior to finalizing the survey instrument (Appendices).

Survey distribution and population

The survey instrument was distributed via the international listserv for pediatric gastroenterology providers maintained by the University of Vermont (pedgi@list.uvm.edu). This listserv helps pediatric gastroenterology providers from all over the world communicate. The survey was sent to 2977 members of the listserv. The survey was open to respondents between 4/13/2022 and 5/12/2022. A total of four emails, one each week of the four weeks it was open, were sent to remind members to respond to the study.

Data analysis

Survey data were stored in REDCap, managed by Stony Brook University. Data analyses were conducted in Stata 16 (StataCorp LLC, College Station, TX). Univariate statistics were calculated for all study variables. Only completed surveys were utilized in the analysis. Chi-squared test was used to assess differences in compliance with European/North American *H. pylori* guidelines and provider or practice characteristics.

## Results

At the completion of the study, 302 individuals completed the survey, representing a response rate of 10.1%. Participants represented pediatric gastroenterology providers from 25 different countries (Table 1). The demographics of respondents (Table 2) show that the majority of respondents were attending physicians and worked at academic medical centers.

**Table 1:** The number of respondents for the survey from each country.

Countries	Number of respondents
Armenia	1
Australia	6
Bangladesh	1
Belgium	1
Bulgaria	1
Canada	9
Chile	1
Colombia	1
Ecuador	1
Egypt	2
India	1
Israel	7
Italy	3
Jamaica	1
Jordan	1
Mexico	4
Netherlands	1
New Zealand	2
Pakistan	2
Portugal	3
Romania	1
Saudi Arabia	1
United Arab Emirates	1
United Kingdom	2
United States of America	236
Undisclosed	12

**Table 2:** Demographics of survey respondents by provider type, years of practice, and practice setting.

Provider type	Respondents	Percentage of total
Fellow/trainee	53	17.5%
Attending physician	221	73.2%
Advanced practitioner	26	8.6%
Undisclosed	2	0.01%
Years of practice	Respondents	Percentage of total
1-5 years	110	36.4%
6-10 years	70	23.2%
11-15 years	45	14.9%
15+ years	75	24.8%
Undisclosed	2	0.7%
Practice setting	Respondents	Percentage of total
Academic medical center	230	76.2%
Community hospital	48	15.9%
Private practice	22	7.3%
Undisclosed	2	0.7%

Univariate results are summarized in Tables 3, 4. There was no statistically significant difference (p = 0.32) in the academic versus non-academic providers with regard to utilizing non-invasive tests first-line. In addition, with regard to the use of non-invasive testing, no difference was seen among more junior practitioners (1-10 years in practice) versus those with 11 or more years in practice (p = 0.88). A statistically significant difference (p = 0.013) was found between practitioners from NASPGHAN countries versus non-NASPGHAN countries regarding the use of upper endoscopy as a first-line diagnostic test, with 173 (70.68%) of NASPGHAN providers diagnosing *H. pylori* by invasive test versus 21 (51.22%) of providers from non-NASPGHAN countries. Overall, providers were more likely to utilize endoscopy as a first-line test if a patient was referred to a provider with a positive non-invasive test. A total of 160 (53.51%) of providers would perform upper endoscopy, 132 (44.15%) would treat *H. pylori*, and seven (2.34%) would repeat *H. pylori* testing.

**Table 3:** Summarization of survey answers. *** Indicates a 2016 guideline recommendation. **** Indicates 2016 guidelines recommended diagnosis of *H. pylori* infection based on either (a) *H. pylori* gastritis plus at least one other positive biopsy-based test or (b) positive culture. PPI: proton pump inhibitor.

Question	Respondents	Percentage of total
What is your first-line test when you suspect a patient has *H. pylori*?		
Stool antigen test	171	56.6%
Upper endoscopy***	97	32.1%
Urease breath test	32	10.6%
Serum antigen test	2	0.7%
If a patient is referred to you for consultation with a positive *H. pylori* breath or stool antigen test, what is your next step?		
Perform upper endoscopy	160	53.5%
Treat *H. pylori* infection	132	44.1%
Does your hospital/center have the ability to check *H. pylori* antimicrobial susceptibility?		
In-house test available	71	23.8%
Send out the test available	130	43.5%
Test is not available	61	20.4%
Unsure	37	12.4%
What is the minimum time you wait after discontinuation of antibiotics prior to testing for *H. pylori*?		
2 weeks	45	14.9%
4 weeks***	181	60.1%
6 or more weeks	75	24.9%
What is the minimum time you wait after discontinuation of acid-suppressing medication prior to testing for *H. pylori*?		
0 weeks	16	5.3%
2 weeks***	141	47.0%
4 weeks	115	38.3%
6 or more weeks	28	9.3%
If you do not have *H. pylori* susceptibility testing results, what is your first-line treatment?		
PPI-amoxicillin-clarithromycin	121	40.5%
PPI-amoxicillin-metronidazole	66	22.1%
PPI-high-dose amoxicillin-metronidazole***	79	26.4%
Bismuth-PPI-amoxicillin-metronidazole	24	8.0%
Bismuth-PPI-metronidazole-tetracycline	9	3.0%
In your practice, how do you diagnose *H. pylori* on endoscopy?****		
Positive bacterial culture	2	0.7%
*H. pylori* gastritis on histopathology	149	49.3%
*H. pylori* gastritis on histopathology with positive bacterial culture***	36	11.9%
*H. pylori* gastritis on histopathology with rapid urease test***	81	28.8%
*H. pylori* gastritis on histopathology with PCR***	3	1.0%
*H. pylori* gastritis on histopathology with fluorescent in situ hybridization***	20	6.6%

**Table 4:** Summarization of survey answers. *** Indicates a 2016 guideline recommendation. **** Guideline recommendation is to discuss the pros and cons of treating, and that *H. pylori* is unlikely cause of the patient’s symptoms. Shared decision-making is to be used to determine treatment decisions.

Question	Frequency, n (%)				
	Never	Not often	Sometimes	Very often	Always
How often do you treat *H. pylori* based on a positive non-invasive test?	48 (15.9%)***	75 (24.8%)	69 (22.8%)	73 (24.2%)	37 (12.3%)
If you are suspicious of *H. pylori* during endoscopy, how often do you send culture and susceptibility for *H. pylori*?	71 (23.5%)	78 (25.8%)	52 (17.2%)	37 (12.2%)	64 (21.2%)***
In the absence of peptic ulcer disease on endoscopy, but histopathology shows *H. pylori* infection, how often would you treat a patient for *H. pylori*?****	2 (0.7%)	21 (6.9%)	46 (15.3%)	94 (31.2%)	138 (45.9%)
How often do you test for *H. pylori* eradication after treatment?	1 (0.3%)	24 (7.9%)	32 (10.6%)	49 (16.2%)	196 (65.9%)***

In response to a question about the availability of *H. pylori* culture and antimicrobial susceptibility testing, it was found that 71 (23.75%) providers have access to in-house culture and susceptibility testing, while 130 (43.48%) had this available as a send-out test. It was also found that 61 (20.4%) of respondents did not have culture and susceptibility testing for *H. pylori* available, and 37 (12.37%) were unsure if this test was available in their practice.

There was a range of responses with regards to how often one sends *H. pylori* culture and susceptibility testing if suspicious for *H. pylori* infection (Table 4). A statistically significant difference was seen between academic and non-academic providers’ likelihood of sending culture and susceptibility testing when suspicious for *H. pylori* infection, with academic clinicians more likely to send antimicrobial susceptibility testing (p = 0.002). In addition, a statistically significant difference was seen with the use of culture and susceptibility testing in the respondents who had access to in-house testing and those who did not have in-house testing (p < 0.001), favoring those who had in-house testing available.

Survey results showed that during endoscopy, most providers (n = 194, 64.45%) took biopsies from both the gastric antrum and gastric body, while 54 (17.94%) providers took from antrum alone and four (1.33%) providers took from the corpus alone while 44 (14.62%) providers took biopsies from antrum, corpus, and fundus of the stomach.

The survey also assessed whether providers check for eradication after completion of *H. pylori* treatment and what test providers utilize to test for *H. pylori* eradication. This showed the majority (n = 196, 64.9%) of respondents always tested for *H. pylori* eradication after treatment, with non-invasive testing of the urease breath test (n = 68, 22.52%) or stool antigen test (n = 225, 74.5%) being the most commonly used methods. Very few respondents (n = 4, 1.32%) utilized upper endoscopy to confirm eradication of the infection.

## Discussion

To our knowledge, this is the largest survey to assess pediatric gastroenterology providers’ adherence to an international guideline by their societies regarding the diagnosis and treatment of *H. pylori* infection. The survey assessed 302 pediatric gastroenterology providers, about 10.2% of the members of the pediatric gastroenterology international listserv. The survey was able to assess the practice styles of providers from 25 different countries; however, the majority of the respondents were from the United States of America (78.14%). Though there has been a recent update to the ESPGHAN/NASPGHAN guidelines in 2024, the majority of the new recommendations reaffirmed the 2016 recommendations; however, antimicrobial regimens were changed [1,5].

This study found that there were areas where adherence to the 2016 guidelines was strong and other areas where adherence to the guidelines was limited.

Use of non-invasive *H. pylori* testing

Guidelines do not recommend non-invasive testing utilizing *H. pylori* stool antigen or urease breath testing [1,5]. The guidelines suggest that a thorough investigation should be performed for a child’s symptoms, and an upper endoscopy is the gold standard test used to evaluate for symptomatic *H. pylori* infection [1,5].

Our study found that two-thirds of providers utilized non-invasive testing (stool antigen or urease breath test) as a first-line test when suspicious of *H. pylori* infection. The most commonly used test is the stool antigen test (n = 171, 56.52%). If a patient was sent for a consultation with a positive test, providers were relatively evenly divided between treating based on outside test result (n = 132, 44.15%) or performing upper endoscopy to confirm (n = 160, 53.51%) the diagnosis. It was found that 110 (36.46%) providers stated that they always or very often treat *H. pylori*, versus 123 (40.72%) providers who said they never or would not often treat *H. pylori* based on a positive non-invasive test. These results were interesting as the ESPGHAN/NASPGHAN do not recommend utilizing non-invasive testing to diagnose or treat pediatric patients with suspected *H. pylori* infection. Our results suggest that a significant portion of clinical providers do not routinely follow these guidelines.

Post-hoc analysis did not find any significant difference in years of practice between junior practitioners (1-10 years in practice) versus senior practitioners (11 or more years in practice) (p = 0.88). There was also no significant difference between gastroenterologists in an academic versus non-academic setting with regard to the use of non-invasive testing (p = 0.32).

Though our study did not assess for reasons why pediatric gastroenterology providers deviate from guidelines, we postulate the following potential reasons. It is possible that providers do not agree with the ESPGHAN/NASPGHAN guidelines for a variety of reasons. The reasons providers may choose non-invasive testing and treatment based on non-invasive testing may include the following: *H. pylori* may increase the risk of gastric ulcerations and gastric carcinoma in the future; the additional cost and risk of endoscopy to diagnose *H. pylori*; parental preferences; or lack of awareness of guidelines.

Use of culture and susceptibility testing

The ESPGHAN/NASPGHAN guidelines recommend that if a provider is suspicious of *H. pylori* infection, the provider should send *H. pylori* culture and susceptibility during an upper endoscopy [1,5].

Our study shows that the majority of centers cannot perform in-house culture and susceptibility testing. Only 71 (23.75%) of providers worked at a center with in-house testing available, and 61 (20.4%) providers had no access to this type of testing. Not surprisingly, providers who worked at a location that provided in-house testing were more likely to send culture and susceptibility testing (p < 0.001). Interestingly, a higher proportion of providers practicing in non-NASPGHAN countries had the availability of in-house culture/susceptibility testing (p = 0.036).

This lack of access to in-house *H. pylori* culture and antimicrobial susceptibility testing represents a significant structural barrier to guideline-concordant care. While the ESPGHAN/NASPGHAN guidelines appropriately emphasize culture-directed therapy to optimize eradication rates and support antimicrobial stewardship, limited laboratory infrastructure may hinder their implementation in many clinical settings. Given the global prevalence of *H. pylori* infection, rising antimicrobial resistance, and the importance of targeted therapy to achieve successful eradication, future studies are needed to better understand the institutional, logistical, and economic factors contributing to the limited availability of in-house testing and to explore strategies for increasing access to *H. pylori* culture and susceptibility testing across pediatric centers.

Use of endoscopy

The ESPGHAN/NASPGHAN also provides recommendations on how many gastric biopsies and where biopsies should be taken to diagnose *H. pylori*. The recommendation is to biopsy both the gastric antrum and the gastric body [1]. Only 194 (64.45%) of respondents biopsied both of these areas.

Diagnosis of *H. pylori*

The ESPGHAN/NASPGHAN guidelines also stipulate that for a biopsy-proven diagnosis of *H. pylori*, a provider requires either (1) positive bacterial culture or (2) *H. pylori* gastritis on histopathology with a secondary test such as positive bacterial culture, rapid urease test, *H. pylori* by polymerase chain reaction, or by fluorescent in-situ hybridization [1]. Pediatric gastroenterologists appear to be divided in their practice style in following this recommendation. *H. pylori* was diagnosed by finding *H. pylori* gastritis on histopathology by 149 (49.34%) of respondents. Only 140 (47.01%) of respondents diagnosed *H. pylori* following the NASPGHAN/ESPGHAN guidelines (Table 4).

Test of cure

The ESPGHAN/NASPGHAN guidelines provide recommendations on the minimum time to wait after discontinuation of antibiotic therapy and acid suppressive therapy prior to sending a test of cure for *H. pylori* to increase the test’s sensitivity. The guidelines recommend waiting at least four weeks after completing antibiotic therapy prior to checking the test of cure for *H. pylori* and recommend waiting at least two weeks after discontinuation of acid-suppressing medication [1,5]. The majority (Table 3) of pediatric gastroenterologists follow minimum-time recommendations.

Treatment decisions

The ESPGHAN/NASPGHAN guidelines suggest only treating children with positive *H. pylori* on endoscopic biopsies, as discussed above, with peptic ulcer disease (PUD). In the absence of PUD, the guidelines recommend discussing with the family and older children that treatment may not resolve abdominal complaints [1,5]. In addition, it is recommended to discuss the risks of both longstanding, untreated *H. pylori* and the risks of treatment. Risks of longstanding *H. pylori* include progression to gastric cancer in adulthood or development of PUD, among other risks. Risks of treatment include the potential to cause changes in the microbiome, risk of treatment failure, and adverse effects of antimicrobial therapy [1].

Respondents of this survey largely did not follow the above recommendation of the ESPGHAN/NASPGHAN. A total of 232 (77.08%) respondents stated that they either very often or always treat *H. pylori* in the absence of PUD (Table 4). We postulate that this may be due to the following reasons: (1) difficulty in convincing parents that the incidental finding of *H. pylori* is not causing the patient’s symptomology; (2) the potential benefit of resolving symptomology with treatment; and (3) the fear that the child may develop complications of chronic *H. pylori* infection in the future.

Antimicrobial therapy

The ESPGHAN/NASPGHAN *H. pylori* guidelines recommend treating the infection based on susceptibility test results [1,5]. It should be noted that there are differences in the 2016 and 2024 recommendations for antimicrobial treatment.

Both guidelines offer a recommended antimicrobial strategy when susceptibility testing is unavailable. Worldwide, clarithromycin resistance has been increasing over the allowable resistance rate threshold of 15% [1]. Therefore, if it is unknown whether a strain of *H. pylori* is susceptible to clarithromycin, it is recommended to avoid regimens containing this antibiotic. The 2016 pediatric guidelines suggest utilizing proton pump inhibitor (PPI) with high-dose amoxicillin and metronidazole for susceptibility-unknown *H. pylori* infections to improve the eradication rate [1]. Only 26.42% (n = 79) of the surveyed population would choose the recommended therapy as a first-line treatment. The majority of providers (n = 121, 40.47%) chose PPI with amoxicillin and clarithromycin regimen. To note, this was the recommended treatment in the 2011 guidelines statement from the ESPGHAN/NASPGHAN, indicating that providers have not kept up to date on new guidelines [6]. The updated 2024 guidelines maintain the same antibiotics for susceptibility-unknown *H. pylori* infection, but also add additional bismuth into the treatment regimen [5].

Post-hoc analysis demonstrated that providers with more experience (11 or more years of practice) were more likely to prescribe clarithromycin-based treatment regimens compared to their junior (1-10 years of practice) counterparts (p = 0.034). These data indicate that it is possible that more experienced practitioners may either be more comfortable treating with clarithromycin-based therapy, or may be unaware of updated guidelines.

The ESPGHAN/NASPGHAN recommends that all patients be tested with non-invasive *H. pylori* tests to verify eradication (test of cure) after treatment of *H. pylori* infection [1,5]. Surprisingly, only 196 (64.9%) of gastroenterologists stated that they always perform a test of cure. When gastroenterologists test for eradication, 97% (n = 298) test with non-invasive testing using stool antigen or urease breath test.

Limitations

This study is a cross-sectional study of pediatric gastroenterology providers assessing adherence to well-established society guidelines. Though our response rate was 10.1% (302 participants), which is the largest survey of *H. pylori* practices in pediatric gastroenterologists, sampling bias is possible. It is possible that the results of the survey would change if there were higher rates of participation from a more diverse group. In addition, survey studies assess what people say they do versus how they actually perform, resulting in recall bias. Social desirability bias, when people answer questions based on how they believe society should answer the question, is also a possibility. However, we believe social desirability bias is low in this study due to it being an anonymous study and also one that is conducted privately online.

Another limitation of this study is that though it is a study of international pediatric gastroenterology providers, providers outside of the United States of America consisted of only 21.85% (n = 66) of the study population. This skews the results to reflect mostly North American practices in treating *H. pylori* infection.

Finally, since the completion of this survey in 2022, new ESPGHAN/NASPGHAN guidelines have been introduced in 2024. Though most of the new recommendations reaffirmed the 2016 recommendations, antimicrobial regimens were changed. Despite new recommendations being released, we believe our study still has relevance as it shows that even six years after publication of the 2016 guidelines, adherence to key recommendations by providers was poor. Our hope is that this survey will highlight the need for more education of pediatric gastroenterology providers on updated recommendations and help medical professionals determine ways to help implement this.

## Conclusions

This study has assessed the reported practice styles of pediatric gastroenterology providers internationally regarding adherence to the ESPGHAN/NASPGHAN *H. pylori* guidelines. There are areas where gastroenterologists have high adherence to the ESPGHAN/NASPGHAN recommendations, which include time to wait before test of cure after stopping antibiotics/acid-suppressing therapy and testing for eradication. However, on issues that are more important to the diagnosis and treatment of *H. pylori*, there seems to be a lack of adherence. Pediatric gastroenterologists had less adherence with the following guidelines: the use of non-invasive testing for the diagnosis of *H. pylori*, treatment based on non-invasive test, appropriate diagnosis of *H. pylori* by endoscopy, utilizing culture and susceptibility testing, and treatment decisions about *H. pylori*. This study also found that the availability of in-house culture and susceptibility is a limiting factor in utilizing this crucial and recommended test, which helps improve eradication rates due to the ability to use targeted therapy.

Pediatric gastroenterologists generally do not adhere well to the ESPGHAN/NASPGHAN guidelines for the testing, diagnosis, or treatment of *H. pylori* infection. This indicates that pediatric gastroenterologists may require further education to raise awareness of guidelines, especially now that there is updated guidance from 2024. Improving adherence to the ESPGHAN/NASPGHAN *H. pylori* guidelines will likely require targeted and pragmatic implementation strategies beyond passive dissemination. Educational efforts could include focused continuing medical education modules, incorporation of guideline-based clinical decision support within electronic medical records, and quality improvement initiatives. In addition, improving adherence to *H. pylori* will likely require a combination of educational, structural, and system-level interventions rather than education alone. Such approaches may help translate updated guideline recommendations into routine clinical care and reduce variability in the diagnosis and management of pediatric *H. pylori* infection.

## Appendices

**Table 5:** Survey questions. NP: nurse practitioner; PA: physician assistant; PPI: proton pump inhibitor.

(1) Please choose the group that best describes you:							
	a. Fellow/trainee						
	b. Attending physician						
	c. Advanced practitioner (for example, NP or PA)						
(2) How many years have you been practicing pediatric gastroenterology?							
	a. 1-5 years						
	b. 6-10 years						
	c. 11-15 years						
	d. More than 15 years						
(3) What is your primary practice setting?							
	a. Academic medical center						
	b. Community hospital						
	c. Private practice						
(4) What country do you practice in?							
	a. Free text						
(5) What is your first-line test when you suspect a patient has H. pylori?							
	a. Stool antigen test						
	b. Urease breath test						
	c. Upper endoscopy						
	d. Serum antigen test						
(6) If a patient is referred to you for consultation with a positive H. pylori breath or stool antigen test, what is your next step?							
	a. Repeat non-invasive testing						
	b. Treat H. pylori infection						
	c. Perform upper endoscopy						
(7) How often do you treat H. pylori based on a positive non-invasive test?							
	a. Never						
	b. Not often						
	c. Sometimes						
	d. Very often						
	e. Always						
(8) If you are suspicious of H. pylori during endoscopy, how often do you send culture and susceptibility testing for H. pylori?							
	a. Never						
	b. Not often						
	c. Sometimes						
	d. Very often						
	e. Always						
(9) Does your hospital/center have the ability to do H. pylori antimicrobial susceptibility?							
	a. Yes, in-house susceptibility is available						
	b. Yes, it is a send-out test						
	c. No						
	d. Unsure						
(10) What is the minimum time you wait after discontinuation of antibiotics prior to testing for H. pylori?							
	a. 0 weeks						
	b. 2 weeks						
	c. 4 weeks						
	d. 6 or more weeks						
(11) What is the minimum time you wait after discontinuation of acid-suppressing medication prior to testing for H. pylori?							
	a. 0 weeks						
	b. 2 weeks						
	c. 4 weeks						
	d. 6 or more weeks						
(12) In your practice, how do you diagnose H. pylori on endoscopy?							
	a. Positive bacterial culture						
	b. H. pylori gastritis on histopathology						
	c. H. pylori gastritis on histopathology with positive bacterial culture						
	d. H. pylori gastritis on histopathology with rapid urease test						
	e. H. pylori gastritis on histopathology with PCR						
	f. H. pylori gastritis on histopathology with fluorescent in situ hybridization						
	g. Unsure						
(13) When evaluating for H. pylori on endoscopy, where do you take biopsies from?							
	a. Antrum						
	b. Body/corpus						
	c. Fundus						
	d. A & B						
	e. A, B, & C						
(14) A patient has an absence of peptic ulcer disease on endoscopy, but histopathology shows H. pylori infection. How often would you treat this patient for H. pylori infection?							
	a. Never						
	b. Not often						
	c. Sometimes						
	d. Very often						
	e. Always						
(15) If you do not have H. pylori susceptibility testing, what is your first-line treatment?							
	a. PPI-amoxicillin-clarithromycin						
	b. PPI-amoxicillin-metronidazole						
	c. PPI-high-dose amoxicillin-metronidazole						
	d. Bismuth-PPI-amoxicillin-metronidazole						
	e. Bismuth-PPI-metronidazole-tetracycline						
(16) How often do you test for H. pylori eradication after treatment?							
	a. Never						
	b. Not often						
	c. Sometimes						
	d. Very often						
	e. Always						
(17) What test do you use to test for H. pylori eradication after treatment?							
	a. Urease breath test						
	b. Stool antigen Test						
	c. Serum antigen Test						
	d. Upper endoscopy with biopsies						
